# Effects of N-acetylcysteine, oral glutathione (GSH) and a novel sublingual form of GSH on oxidative stress markers: A comparative crossover study.

**DOI:** 10.1016/j.redox.2015.07.012

**Published:** 2015-07-29

**Authors:** Bernard Schmitt, Morgane Vicenzi, Catherine Garrel, Frédéric M. Denis

**Affiliations:** a Centre d’Enseignement et de Recherche en Nutrition Humaine, Centre Hospitalier de Bretagne Sud, 5 Avenue De Choiseul, BP 12233, 56322 Lorient Cedex, France; b Laboratoires Le Stum, 4 impasse de Kerhoas, 56260 Larmor Plage, France; c Unité de Biochimie Hormonale et Nutritionnelle, Département de Biochimie, Toxicologie et Pharmacologie, Institut de Biologie et de Pathologie, Centre Hospitalier Universitaire de Grenoble, CS10217, 38043 Grenoble, France

**Keywords:** Glutathione, Oral bioavailability, N-acetyl cysteine, Oxidative stress, Metabolic syndrome, Dietary supplement, Nutraceuticals, Sublingual

## Abstract

Glutathione (GSH) is critical to fight against oxidative stress. Its very low bioavailability limits the interest of a supplementation. The purpose of this study was to compare the bioavailability, the effect on oxidative stress markers and the safety of a new sublingual form of GSH with two commonly used dietary supplements, N-acetylcysteine (NAC) and oral GSH. The study was a three-week randomized crossover trial. 20 Volunteers with metabolic syndrome were enrolled. GSH levels and several oxidative stress markers were determined at different times during each 21-days period. Compared to oral GSH group, an increase of total and reduced GSH levels in plasma and a higher GSH/GSSG ratio (*p*=0.003) was observed in sublingual GSH group. After 3 weeks of administration, there was a significant increase of vitamin E level in plasma only in sublingual GSH group (0.83 µmol/g; *p*=0.04). Our results demonstrate the superiority of a new sublingual form of GSH over the oral GSH form and NAC in terms of GSH supplementation.

## Highlights

• Oral GSH is known for its poor bioavailability in humans.
• We compared a new sublingual GSH with NAC and oral GSH on oxidative stress markers.
• On a 3-weeks period, sublingual GSH improved the GSH/GSSG ratio.
• Sublingual GSH significantly increased plasmatic vitamin E levels in subjects.
• The sublingual form of GSH showed superior effects over NAC and oral GSH.

## Graphical abstract

**Figure:**
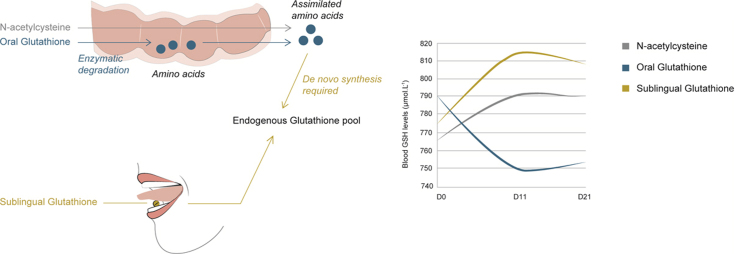

## 1 Introduction

Glutathione (GSH) is an ubiquitous water-soluble molecule found in millimolar concentration in many tissues and cells. It is the most abundant intracellular low molecular weight peptide containing a thiol group. This thiol function is critical for the biological activity of GSH [1].

GSH is made from three amino acids: glycine, cysteine and glutamic acid. This tripeptide exists in reduced (GSH) and oxidized (GSSG) forms. The relative amounts of each form determine the cellular redox status (GSH/GSSG ratio) which is often used as a marker of antioxidative capacity of cells [2].

GSH exhibits diverse physiological roles. It is a potent free radical and reactive oxygen species scavenger [3]. It reacts with various molecules (metabolites, xenobiotics) to form conjugates [4]. GSH functions as a thiol buffer for many cellular proteins (metallothioneins, thioredoxins). GSH is an essential cofactor for many enzymes and it is involved in several metabolic and signaling pathways [5]. GSH is also critical for the regeneration of other antioxidants such as tocopherols and ascorbate [6].

There is growing evidence that dysfunctional GSH homeostasis is implicated in the etiology of several diseases. The most well-known conditions associated with GSH depletion include neurodegenerative diseases [7,8], pulmonary diseases [9], liver diseases [10], immune disorders [11], cardiovascular diseases [12,13] as well as the aging process itself [14].

Several studies showed that plasma GSH levels decrease with age. This deterioration of GSH homeostasis could participate, with other physiological events, in the ageing process and the appearance of age-related diseases[15].

Thus, dietary supplementation with antioxidants has been studied extensively as a potential way to prevent these diseases by countering the negative effects of oxidative stress.

Researchers suggest that GSH is poorly absorbed by oral route mainly due to the action of an intestinal enzyme, the γ-glutamyl transpeptidase (GGT) which degrades GSH[16]. Several studies showed that GSH supplementation in animals is effective, with benefits in the enhancement of immune function[17]; protection against the carcinogenesis process[18]; and improvement of the elimination of toxic chemicals[19].

However, in humans, the effectiveness of an oral supplementation with GSH is very controversial. A number of studies were conducted but using intravenous or nebulized GSH. The results of studies conducted with oral GSH are mixed [20,21]. This differential absorption of oral GSH between humans and rats/mice could be explained by a difference in quantity and activity of the intestinal GGT enzyme [21].

Although oral GSH appears to be the most convenient and safe way to take GSH, the lack of efficacy explains why this form is not often used in clinical trials.

Another strategy to enhance GSH production relies on the use of N-acetylcysteine (NAC), a cysteine precursor. Indeed, the amino acid cysteine is the main factor limiting the synthesis of GSH. Several studies showed that NAC is well absorbed by the intestine and that a supplementation with NAC is effective for increasing GSH levels [22]. However, supplementing with NAC relies upon the body's ability to synthesize glutathione from available raw materials, an ability that diminishes with age and in the presence of certain diseases, especially dysfunction of the liver.

Finding a way to safely, conveniently and rapidly improve GSH levels by directly delivering the whole GSH molecule in its active reduced form would be medically compelling.

Because oral GSH is enzymatically degraded in the intestine, one solution could be using a sublingual delivery system for GSH. Argument for that delivery route is that the GSH tripeptide is very well absorbed through mucosa. It is also well-known that sublingual route allows to by-pass the effect of hepatic first-pass metabolism. In order to address this hypothesis, a food-grade sublingual dosage form was designed.

The purpose of this study was to compare the use of this novel sublingual form of GSH with two commonly used dietary supplements, NAC and oral GSH, to determine their respective interest for raising GSH levels and acting on the GSH/GSSG ratio.

## 2 Experimental section

This randomized crossover study focused on the efficiency of a sublingual form and a conventional oral form of GSH compared to effects of a precursor (NAC) taken as a baseline. The evaluation focused on the bioavailability of both GSH forms, their respective effect on oxydative stress markers, and their tolerability.

### 2.1 Participants

The study protocol was reviewed and approved by ethics committee (CPP Ouest) and the Agence Nationale de la Sécurité du Médicament (ANSM). The approval code of this study is AEC/B120599-40. All clinical activities were conducted at the Centre Hospitalier Bretagne Sud (Lorient, France), in accordance with the Helsinki Declaration, the French Public Health Code concerning the biomedical researches and the rules of Clinical Best Practice.

A total of 20 voluntary subjects (5 men and 15 women) was enrolled. Baseline characteristics of the participants are given in Table 1 below.

Regarding the inclusion criteria, all subjects had risk factors of low-grade inflammatory state corresponding to metabolic syndrome as defined in the guidelines of the National Cholesterol Education Program ATP III[23]: abdominal obesity associated with at least two of the following factors: impaired fasting glucose with glucose levels between 1.10 and 1.27 g/l (≥6 and <7 mmol/l), elevated blood pressure (SBP >130 mm Hg and/or DBP >85 mm Hg), hypertriglyceridemia >1.45 g/l (≥1.65 mmol), LDL-C between 1.6 and 2.2 g/l (4.13 and 5.68 mmol/l), HDL-C <0.40 g/l (1 mmol/l) (male) and 0.50 g/l (1.3 mmol/l) (female). They had a low tobacco consumption (<5 cigarettes/day) associated with a sedentary lifestyle.

Regarding the exclusion criteria, the subjects took no anti-inflammatory or non-steroidal anti-inflammatory drugs. They had no previous history of cardiovascular disease or symptomatic chronic inflammatory disease. They did not consume antioxidant supplements or cholesterol-lowering agents. They had a free regular diet respecting their family habits, but neither limited nor extensive (average energy: 2500 kcal/day) and a moderate alcohol intake (<20 g/day) according to the criteria of the Alcohol Use Disorder Identification Test. GGT were inferior to 35 IU/l and Carbohydrate Deficient Transferrin (CDT) inferior to 3%.

20 Participants completed the study and all the data were analyzed.

### 2.2 Products tested

#### 2.2.1 Composition of the sublingual form of glutathione

This new and patented sublingual form of GSH (Sublinthion®) was provided by Laboratoires Le Stum (Larmor-Plage, France). One sublingual tablet contains 150 mg of reduced GSH. The dosage was one tablet 3 times per day (in the morning, the midday and the evening), to let melt under the tongue. It represents a daily intake of 450 mg of GSH.

#### 2.2.2 Composition of the oral form of glutathione

The oral form of reduced GSH was l-Glutathione Reduce® (Laboratoire Equi-Nutri, Belgium). One capsule contains 150 mg of GSH. The dosage used in the study was one capsule 3 times per day providing a total intake of 450 mg of GSH.

#### 2.2.3 Composition of the precursor NAC taken orally

The NAC drug used for this study was marketed under the brand name Fluimucil® (Laboratoires Zambon, France). A sachet contains 200 mg of NAC. The dosage was one sachet per day.

Cysteine is the limiting factor for GSH synthesis and its represents 33.6% of the GSH molecule [24]. Providing 200 mg of cysteine (commercial dosage) would be sufficient for the body to theoretically synthetize de novo up to 600 mg of GSH.

### 2.3 Study design

Each product was administered successively during three periods (P1, P2 and P3) of 21 days each. A wash-out period of 14 days was observed between each product. Six combinations of administration were possible: [NAC-PO-SL]; [NAC-SL-PO]; [PO-SL-NAC]; [PO-NAC-SL]; [SL-PO-NAC]; [SL-NAC-PO] (PO=oral form of GSH; SL= sublingual form). Each combination was randomly assigned to volunteers at the inclusion, by respecting a minimum of three subjects per combination. Each period included three visits: V1; V2 (V1+10±2 days) and V3 (V2+10±2 days). In total, each volunteer received two preliminary visits (pre-inclusion and inclusion) and nine visits during the protocol.

During the pre-inclusion visit, inclusion/exclusion criteria were explained to subjects, the initial screening was done according to these criteria and the informed consent of each subject was collected.

A full medical history from each participant was obtained during the inclusion visit. Medical examinations were also conducted to check the digestive, cardiovascular and pulmonary status of the subjects.

**Fig. 1:**
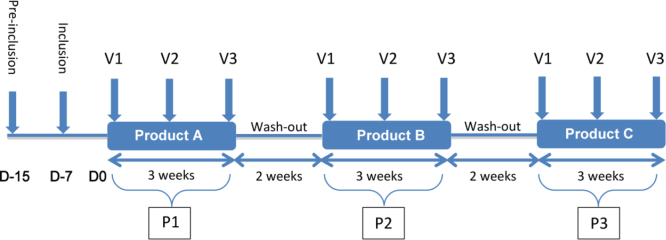
Design of the study.

The study was conducted according to the following plan (Fig. 1).

During the test there was no change of the diet habits or the lifestyle of the participants (diet, physical activity, smoking, etc.). Each patient was his own control to avoid a potential bias of a residual effect of the taking of the previous product on the following product. Table 2 presents the average characteristics for each group at the time of inclusion in the study.

### 2.4 Markers and parameters measured

Blood tests were realized at the beginning (visit 1, before taking treatment), at the middle (visit 2) and at the end (visit 3) of every period, for all the volunteers. At each visit, the following parameters were measured.

#### 2.4.1 Total GSH; GSH (reduced); GSSG (oxidized); ratio GSH/GSSG

Endogenous glutathione was measured according to the following analytical protocol. Briefly, arterial blood samples were collected in lithium heparin vacutainers as an anticoagulant. 400 µl of whole blood were collected in 3.6 ml of metaphosphoric acid for the determination of total, reduced (GSH) and oxidized (GSSG) glutathione. After centrifugation (4000*g*, 10 min, 4 °C) total and reduced GSH were determined enzymatically in the acidic protein-free-supernatant. The assay of GSSG was performed after having masked GSH by adding 2-vinylpyridine to the deproteinized extract and also determined enzymatically[25].

#### 2.4.2 Reduced thiols

The rest of whole blood was centrifuged (4000 g, 10 min, 4 °C) to separate the plasma for thiols, Plasma was collected in Eppendorff sterile tubes and stored at -80 °C until assayed. The measurement of thiol groups was performed using Ellman's reagent[26,27]. Briefly, 5,5′-dithio-bis (2-nitrobenzoic acid) 2.5 mM in 0.2 M phosphate buffer, pH 8.0 was mixed with 500 µl of sample and 750 µl of 50 mM phosphate buffer 50 mM pH 8.0 and baseline absorbance recorded at 412 nm. Then, 250 µl of freshly prepared Ellman's reagent were added, reaction allowed to proceed for 15 min at room temperature in the dark and final absorbance measured. Thiol values were expressed in µmol/g protein using a molar absorption coefficient of 13,600 l mol^−1^ cm^−1^ for thiol 5,5′-dithio-bis (2-nitrobenzoic acid) complex. A calibration curve was performed by sequential dilution of a 1 mM N-acetyl cysteine stock solution.

#### 2.4.3 Vitamin E: alpha and gamma tocopherols

Serum concentrations of tocopherols were measured by high performance liquid chromatography as previously described[28].

#### 2.4.4 Lipid status: total cholesterol (TC), LDL-C, HDL-C, triglycerides (TG)

Total cholesterol, HDL, and TG were measured by spectrophotometric methods on a routine chemistry system (Vitros Fusion 5.1, Ortho Clinical Diagnostics, USA). Serum LDL-cholesterol was calculated using the following Friedewald formula [29]:

$$\left[\text{LDL−C}\right]=\left[\mathrm{TC}\right]-\left[\text{HDL−C}\right]-0,20^{⁎}[\mathrm{TG}]\;\left(\mathrm{expressed}\;\mathrm{in}\;g/l\right)$$

### 2.5 Statistical analysis

The individual characteristics (age, sex) and all the parameters at D0 are described with regard to the mean and the standard deviation. The conditions of normality are verified in advance by the Shapiro–Wilk test, comparability of groups is performed by Student's *t*-test. From a value of *σ* estimated on the basis of a previous pilot study (internal documents), we used a sample size of 20 volunteers, who were included. To search a “carry-over” effect (i.e. a residual effect of a product of a previous period over the next period), the measures obtained at the end of 3 treatment periods (P1–V3; P2–V3; P3–V3) were compared by using a mixed linear regression model for each of three treatments.

The statistical analysis was performed with the SAS software version 9.3 (SAS Institute Inc., Cary, NC). *P*≤0.05 was taken as the level of statistical significance for all procedures.

### 2.6 Outcomes

The purpose of the study was to compare the bioavailability of each product, the effects of the different forms of glutathione on markers of oxidative stress and the safety of each product.

#### 2.6.1 Bioavailability evaluation

The bioavailability of each product was measured by comparing respective total glutathione (GSHt), reduced glutathione (GSH) and oxidized glutathione (GSSG) indices. The measurements were made by comparing these markers between the first and second visit (V2–V1) and between the first and last visit (V3–V1).

#### 2.6.2 Evaluation of the antioxidant efficacy

The primary outcome was the ratio between reduced glutathione and oxidized glutathione expressed as GSH/GSSG.

The GSH/GSSG ratios were compared at the end of each period by using a mixed linear regression model. The equation is the following one:

[Primaryoutcome(GSH/GSSG)=(a0+ai)+a1*treatment+a2*period+a3*(GSH/GSSHratioatvisit1)

In the equation above, *ai* (*a* index *i*) represents the individual variability, to which the standard intercept (*a*0) is added. To evaluate the effect of each treatment, the model is adjusted to the value of the primary outcome at the start of each treatment period (P1–V1; P2–V1; P3–V1). The model is also adjusted to the treatment period (3 periods for each subject corresponding to the successive intake of the 3 treatments). This adjustment allows to take into consideration a potential effect of time on the primary outcome [30].

The absence of “Carry-Over” effect, whatever the period and the product considered, allowed us to apply the above equation to all parameters studied.

The secondary outcomes contributed to estimate the oxidative stress status of the volunteers: plasma reduced thiols, vitamin E, Total Cholesterol, HDL-C, LDL-C and plasma triglycerides were also measured.

#### 2.6.3 Tolerance of the treatments

The tolerance of the treatments was analyzed by evaluating changes in plasma levels of CRPus (ultra-sensitive C-reactive protein) and liver function tests (Alanine amino transferase (ALAT), Aspartate amino transferase (ASAT), alkaline phosphatase (AP) and GGT between the first visit (V1) and the last visit (V3) for each treatment). The values did not have to exceed the superior limit of the range of normality (*N*): ALAT (*N*: 30–50 IU/l); ASAT (*N*: 15–41 IU/l); AP (*N*: 40–150 IU/l); GGT (*N*: 5–50 IU/l). The percentage increase of these biological data was analyzed using the Wilcoxon test of signed rank.

The evaluation of safety also included the monitoring of any clinical adverse events as well as vital signs (heart rate, blood pressure, respiratory frequency, body temperature). The general appearance was checked by a physical examination of each subject at every visit (V1, V2, V3).

## 3 Results

### 3.1 Bioavailability

The comparative analysis of bioavailability between oral GSH and the sublingual form is summarized in Tables 3 and 4.

The potential problem in crossover design is that carryover effects may bias the direct effects of the treatment. Regardless of the period and the treatment, no significant carryover effect was observed (*p*>0.75). Therefore, the data were pooled for a given treatment (SL, PO) to analyze the results (*n*=20 for each treatment).

### 3.2 Effects of the treatments

#### 3.2.1 Primary outcome (GSH/GSSG)

The comparison of the effect of each treatment on the GSH/GSSG ratio was performed after pooling the results as there was no carryover effect.

The evolution of the GSH/GSSG ratio for the 3 groups (NAC, oral GSH or sublingual GSH) is reported in Table 5.

A comparative analysis was performed: first by taking NAC as reference (comparison NAC vs oral GSH and NAC vs sublingual GSH) and second by comparing the oral GSH to the sublingual GSH form.

In the oral GSH group, the GSH/GSSG ratio was low at each time and significantly different at V3 (*p*=0.03) compared to the NAC group.

In the sublingual GSH group, this ratio tended to be high at each time and was statistically significant at V2 (*p*=0.03) compared to the NAC group.

Compared to the oral GSH group, the sublingual group exhibited a higher GSH/GSSG ratio, in particular at V3 (*p*=0.02).

#### 3.2.2 Secondary outcomes

##### 3.2.2.1 Reduced thiols

Results are detailed in the Table 6. Reduced thiols levels are expressed on an albumin gram basis.

An intragroup analysis was performed: in the NAC group, a significant increase was observed at V2 compared to baseline (0.12 µmol/g, *p*=0.04). In the oral GSH group, the level of reduced thiols increased significantly at V2 and V3 compared to baseline (respectively 0.14 µmol/g; *p*=0.004 and 0.13 µmol/g, *p*=0.001). For sublingual GSH, this level increased only in the first period (0.14 µmol/g, *p*=0.01). No significant differences were observed between the 3 groups.

##### 3.2.2.2 Vitamin E

The effects of the 3 treatments on the levels of vitamin E in plasma were also examined (Table 7).

After 3 weeks of administration, there was a significant increase of vitamin E level in plasma only in the sublingual GSH group (0.83 µmol/g; p=0.04). No significant differences were observed between the 3 groups or for the oral GSH and NAC arms.

##### 3.2.2.3 Lipid status

Results are detailed in the Table 8.

After performing an intragroup analysis, no changes were observed at any time points or in either groups, whatever the lipid biomarker monitored (total cholesterol, HDL-C, LDL-C, TG).

When taking the lipid values of the NAC group as baseline, total cholesterol and LDL-C were slightly decreased at V3 in both oral and sublingual GSH groups. However, it was not statistically significant.

In the meantime, HDL-C level decreased in the oral GSH group and increased in the sublingual GSH group but these differences were not significant. However, compared to the oral GSH group, a significant increase of HDL-C level was observed in the sublingual GSH group (0.039±0.013, p=0.0043).

#### 3.2.3 Adverse effects

Values of the plasma levels of CRPus and liver function markers at each visit for each treatment arm were reported in Table 9.

Whatever the marker (hepatic status or ultra-sensitive CRP), no significant changes were reported. For all the markers monitored, values were always within the range of normality.

All the dosage forms were very well tolerated and no adverse events were reported by the participants, whatever the treatment used.

## 4 Discussion

Conducting such a study is always difficult, as the supplementation product (GSH) is also produced endogenously by the body. Furthermore, like most of our antioxidants, it is tightly regulated.

Whatever the treatment considered, our results sometimes show high standard deviations. This can be explained by the heterogeneity of the studied population. Indeed, the main inclusion criterion was the presence of metabolic syndrome. It is not a strictly defined disease entity, but a convergence of at least 3 risk factors. As each patient may have a number and/or a combination of different risk factors while meeting the strict definition of metabolic syndrome, this heterogeneity was expected. Otherwise, it corresponds to the remaining diversity commonly encountered in a standard population. Therefore, bias in the recruitment of the volunteers can be excluded for this study.

Oral administration of GSH is not considered optimal due to its very poor bioavailability and rapid oxidation. Other indirect means have been developed to circumvent this problem. One of them is the oral delivery of NAC as a source of cysteine. Indeed, after its intestinal absorption, NAC undergoes first-pass metabolism in the liver where it is deacetylated to cysteine. Then, unless there is hepatic dysfunction, the hepatic tissue synthetizes *de novo* GSH from this cysteine. This GSH replenishes the hepatic stock before being released in the plasma [22,31]. Consequently, we considered NAC as the reference treatment for this study.

Regarding the bioavailability of oral GSH, our data are consistent with previous results from a study of oral GSH supplementation (1 g/day for 4 weeks) that showed a non-significant decrease of total and reduced GSH indices [20]. This results in an overall decrease of the GSH/GSSG ratio. High levels of GSSG are indicative of periods of oxidative stress. The ratio GSH/GSSG is an important marker of redox status. The restoration of a normal redox equilibrium results in an increase in GSH/GSSG ratio. In our study, the GSH/GSSG ratios in the NAC and sublingual GSH arms are significantly higher than oral GSH one. It seems that the sublingual GSH form is more useful than the oral form to improve this balance.

One possible explanation of these results is that oral GSH undergoes partial hydrolysis and oxidation during the digestive process. Therefore, the liver has to synthetize de novo GSH from precursors.

With the sublingual dosage form, the GSH is directly assimilated through the buccal mucosa and avoid the hepatic first-pass effect. Our results suggest that the sublingual GSH form exhibits a better bioavailability than the oral GSH.

This increase of the GSH/GSSG ratio may suggest a reduction in oxidative stress resulting from the sublingual GSH supplementation. Therefore, we tried to determine whether the improved bioavailability of the sublingual form of GSH resulted in an effect on oxidative stress markers. The outcome measures were extended to reduced thiols and vitamin E.

Some evidence suggest that GSH is critical for the recycling of antioxidants like vitamin C [32,33] and consequently, vitamin E [34], which is an important inhibitor of the lipid peroxidation. Our findings are consistent with these previous observations. We found that there was a significant increase in the plasma vitamin E level following the supplementation with sublingual GSH.

Considering that GSH forms were given at physiological dose to subjects, obtaining such significant differences between these two dosage forms, either on the primary endpoint or on several secondary endpoints, reinforces the legitimacy of the sublingual form over the oral GSH.

It would be interesting to conduct the same study on a population with greater GSH deficiency or high oxidative stress (smokers, type 2 diabetics, HIV-positive subjects). Larger differences between the two forms of GSH on the GSH/GSSG ratio or on other markers would likely be expected.

Given the results observed between NAC and sublingual GSH, it seems useful to advise this new sublingual form for the same indications as those described for the NAC in the literature [35]. The recommendation of this product should be preferentially based on clinical observation and inventory of risk factors rather than based on costly and variable blood tests.

Regarding the duration of treatment necessary to obtain an antioxidative effect, 21 days of treatment were sufficient to achieve significant results, mostly for the NAC and the sublingual GSH form. During these 3 weeks, no adverse effects were reported. However, the overall duration of treatment must take into account the importance and the multiplicity of risk factors.

## 5 Conclusions

Conducted on a population at risk with metabolic syndrome, the objective of this study was to assess the bioavailability, the effect on biomarkers, and the short-term safety of 2 dosage forms of glutathione, the cornerstone of antioxidant defenses.

Overall, our results demonstrate the significant superiority of a new sublingual form of GSH over the oral form, both in terms of bioavailability and positive effects on oxidative stress. Compared to NAC, better effects of the sublingual form of GSH were also observed.

Metabolic syndrome increases the risk of developing cardiovascular diseases and diabetes. Because of the impact of the deleterious effects of reactive oxygen species (ROS) in the promotion and the development of the metabolic syndrome, it is important to establish a preventive strategy and fight against oxidative stress. In addition to the usual dietary recommendations, it is reasonable to propose to concerned people a product whose interest is demonstrated.

This new sublingual formulation of GSH meets this requirement. It should find its place in the primary and secondary prevention strategies.

## Author contributions

Conceived and designed the experiments: Bernard Schmitt, Morgane Vicenzi and Frederic M. Denis. Performed the experiments: Bernard Schmitt. Analyzed the data: Bernard Schmitt. Contributed reagents/materials/analysis tools: Catherine Garrel. Wrote the paper: Bernard Schmitt, Morgane Vicenzi and Frederic M. Denis. All authors read and approved the final manuscript.

## Conflicts of interest

Frederic M. Denis and Morgane Vicenzi are employees of Laboratoires Le Stum. The other authors declare no conflict of interest.

## Figures and Tables

**Table 1:** Baseline characteristics of study participants (mean±SD).

	Female (*n*=15)			Male (*n*=5)		
	Mean	Max	Min	Mean	Max	Min
AGE (years)	59.73±8.4	67	38	53.6±4.67	59	47
BODY WEIGHT (kg)	75.97±10.17	87.5	52	88.44±5.18	95	84
HEIGHT (cm)	152.30±5.34	168	150	175.20±5.54	182	169
BMI (kg/m^2^)	28.58±2.81	32.42	22.51	28.80±0.75	30	28
SBP (mm Hg)	135±15.3	160	110	129.2±11.6	150	130
DBP (mm Hg)	77.8±11.2	100	60	80.0±8.4	90	65
TRIGLYCERIDES (g/l)	1.27±0.63	2.61	0.44	1.74±0.70	2.68	0.60
LDL-C (g/l)	1.48±0.29	1.97	0.87	1.30±0.41	1.74	0.60
HDL-C (g/l)	0.68±0.28	1.38	0.41	0.65±0.37	1.39	0.36
GLYCEMIA (g/l)	0.95±0.11	1.19	0.81	1.07±0.11	1.27	0.96

BMI: Body Mass Index; SBP: Systolic Blood Pressure; DBP: Diastolic Blood Pressure

**Table 2:** Characteristics of volunteer groups at inclusion (mean±SD).

	Group^a^	Age (years)	Height (m)	Weight (kg)	BMI (kg/m^2^)	Systolic pressure (mm Hg)	Diastolic pressure (mm Hg)
1	NAC-PO-SL	55±15	1.61±0.06	70.9±5.1	27.4±0.8	132±19	77±6
	(*n*=3)						
2	NAC-SL-PO	54±10	1.65±0.06	77.9±7.3	28.6±1.9	138±18	80±9
	(*n*=4)						
3	PO-SL-NAC	63±5	1.61±0.04	75.1±7.4	28.9±2.0	135±14	82±4
	(*n*=4)						
4	PO-NAC-SL	59±8	1.54±0.06	71.4±17.0	30.0±5.4	137±12	83±15
	(*n*=3)						
5	SL-PO-NAC	52±5	1.69±0.09	85.1±10.6	29.6±1.7	138±17	84±13
	(*n*=3)						
6	SL-NAC-PO	61±7	1.63±0.15	76.6±14.3	28.6±2.0	125±9	77±6
	(*n*=3)						
	Comparison groups^b^	*p*=0.26	*p*=0.23	*p*=0.28	*p*=0.59	*p*=0.80	*p*=0.72

a Successive treatment.

b Kruskal–Wallis test.

**Table 3:** Total glutathione, GSH and GSSG levels (µmol/l) (mean±SD).

Product	Dosage	V1	V2	V3	ΔV2–V1	ΔV3–V2	ΔV3–V1
NAC (*n*=20)	Total GSH	800.24±94.87	825.53±127.62	821.0±124.88	30.35±74.73	4.31±47.6	27.0±77.75
	GSSG	17.32±5.24	17.87±4.38	15.60±4.83	0.55±4.03	−1.66±2.91	−7.33±11.0
	GSH	765.59±91.94	789.79±122.83	789.80±119.40	24.21±63.64	11.14±45.80	30.95±71.84
							
PO (*n*=20)	Total GSH	823.29±90.51	782.69±96.89	789.88±133.55	-37.44±72.41	3.19±103.63	-33.41±84.01
	GSSG	16.32±3,48	16.08±4.10	18.60±5.29	−0.06±3.34	2.33±6.32	2.28±4.62
	GSH	790.66±87.99	750.54±96.90	752.68±129.53	−37.58±67,78	-1.46±99.74	−37.98±80.58
							
SL (*n*=20)	Total GSH	811.12±99.77	846.0±127.88	838.76±97.69	34.88±61.52	−7.24±50.57	27.65±57.71
	GSSG	17.61±4.03	16.54±4.70	15.62±3.62	−1.07±4.17	−0.92±4.38	−2.01±4.26
	GSH	774.71±99.45	812.92±122.90	807.53±96.15	38.73±57.96	−5.39±48.25	32.41±57.54

**Table 4:** Evolution of total GSH, GSH and GSSG: oral versus sublingual GSH (µmol/l).

	Dosage		ΔV2–V1	ΔV3–V2	ΔV3–V1
Comparison	Total GSH	PO	−37.44	3.19	−33.41
PO vs SL		SL	34.88	−7.24	27.65
(*n*=20)		*p*	*0.02*	0.37	*0.05*
	GSSG	PO	−0.06	2.33	2.28
		SL	−1.07	−0.92	−2.01
		*p*	0.23	0.12	0.04
	GSH	PO	−37.58	−1.46	−37.98
		SL	38.73	−5.39	32.41
		*p*	*0.01*	0.41	*0.03*

Compared to the oral GSH group, an increase of total and reduced GSH levels in plasma was observed in the sublingual GSH group. The GSSG level also decreased following the supplementation with the sublingual GSH. These differences between the 2 groups were statistically significant (*p*≤0.05), whatever the parameter considered.

**Table 5:** GSH/GSSG ratio and their evolution (mean±SD).

Product	V1	V2	V3	ΔV2–V1	ΔV3–V2	ΔV3–V1
NAC (*n*=20)	50.03±14.02	46.25±7.17	56.44±13.74	−3.79±11.39	9.71±10.81	7.38±11.12
Oral GSH (*n*=20)	51.68±11.04	51.54±14.28	44.76±14.23	−0.91±9.35	−6.31±17.41	−6.92±15.90
Sublingual (*n*=20)	47.55±12.50	53.69±13.84	56.97±16.22	6.15±10.41	3.27±14.75	9.42±14.62
Comparison NAC/PO	*p*=0.28	*p*=0.20	*p=0.03*	*p*=0.29	*p*=0.06	*p=0.01*
Comparison NAC/SL	*p*=0.22	*p=0.03*	*p*=0.37	*p=0.02*	*p*=0.22	*p*=0.20
Comparison PO/SL	*p*=0.11	*p*=0.20	*p=0.02*	*p=0.03*	*p*=0.07	*p=0.002*

**Table 6:** Evolution of reduced thiols (µmol/g) for each treatment (mean±SD).

	V1	V2	V3	Intragroup evolution		
				ΔV2–V1	ΔV3–V1	ΔV3–V2
NAC (*n*=20)	6.24±0.32	6.36±0.32	6.29±0.44	0.12, *p=0.04*	0.05, *p*=0.29	−0.07, p=0.17
Oral GSH (*n*=20)	6.15±0.28	6.29±0.28	6.28±0.38	0.14, *p*=*0.004*	0.13, *p*=*0.001*	−0.01, *p*=0.5
Sublingual GSH (*n*=20)	6.14±0.29	6.28±0.36	6.14±0.33	0.14, *p*=*0.01*	0.00, *p*=0.46	−0.14, *p*=0.07
Comparison NAC/PO	*p*=0.40	*p*=0.40	*p*=0.39			
Comparison NAC/SL	*p*=0.21	*p*=0.20	*p*=0.15			

**Table 7:** Vitamin E levels (µmol/g) and their evolution (mean±SD).

Product	V1	V2	V3	ΔV2–V1	ΔV3–V1	ΔV3–V2
NAC (*n*=20)	26.63±6.02	25.88±6.39	27.16±5.56	−0.75, *p*=0.10	0.53, *p*=0.18	1.28, *p*=0.10
Oral GSH (*n*=20)	26.70±4.94	26.41±5.52	26.23±5.64	−0.29, *p*=0.24	−0.47, *p*=0.15	−0.18, *p*=0.31
Sublingual GSH (*n*=20)	26.59±5.76	26.71±5.92	27.42±6.32	0.12, p=0.44	0.83, *p*=*0.04*	0.71, *p*=0.25
Comparison NAC/PO	*p*=0.32	*p*=0.31	*p*=0.17			
Comparison NAC/SL	*p*=0.49	*p*=0.25	*p*=0.45			

**Table 8:** Lipid biomarkers levels (g/l) and their evolution (mean±SD).

**Product**	**Dosage**	**V1**	**V2**	**V3**
**NAC (*n*=20)**	**TG**	1.52±0.59	1.49±0.67	1.59±0.63
	**TC**	2.29±0.39	2.28±0.45	2.28±0.41
	**HDL-C**	0.52±0.11	0.51±0.1	0.52±0.1
	**LDL-C**	1.48±0.34	1.47±0.4	1.45±0.39
				
**Oral GSH (*n*=20)**	**TG**	1.52±0.58	1.62±0.92	1.54±0.72
	**TC**	2.32±0.38	2.29±0.41	2.23±0.36
	**HDL-C**	0.52±0.1	0.52±0.12	0.51±0.1
	**LDL-C**	1.49±0.33	1.46±0.35	1.41±0.33
				
**Sublingual GSH (*n*=20)**	**TG**	1.7±0.74	1.4±0.61	1.55±0.63
	**TC**	2.3±0.4	2.29±0.39	2.25±0.43
	**HDL-C**	0.51±0.12	0.53±0.12	0.54±0.13
	**LDL-C**	1.45±0.38	1.49±0.35	1.41±0.4

**Table 9:** Biological tolerance of the treatments (mean±SD).

		**V1**	**V2**	**V3**
**NAC (*n*=20)**	**CRPus (IU/l)**	5.2±0.6	6.4±4.3	5.4±1.2
	**ASAT (IU/l)**	19.32±6.87	18±5.02	18.08±5.83
	**ALAT (IU/l)**	24.52±10.26	22.84±10.12	23.64±11.3
	**AP (IU/l)**	60.04±15.44	60.76±15.12	61.76±14.41
	**GGT (IU/l)**	36±24.3	33.88±21.35	35.36±21.85
				
**Oral GSH (*n*=20)**	**CRPus (IU/l)**	5.2±0.6	5.4±1.5	5.3±0.9
	**ASAT (IU/l)**	19.16±6.82	18.29±6.6	20.48±6.63
	**ALAT (IU/l)**	25.16±14.24	23.38±11.34	27.44±12.59
	**AP (IU/l)**	59.16±13.98	61.92±15.18	61.64±14.53
	**GGT (IU/l)**	34.72±22.89	35.42±21.23	38.88±29.21
				
**Sublingual GSH (*n*=20)**	**CRPus (IU/l)**	7±8.4	5.8±2.3	8.8±17.5
	**ASAT (IU/l)**	19.52±8.1	20±9.23	20±7.44
	**ALAT (IU/l)**	24.6±13.6	27.2±18.69	25.75±12.49
	**AP (IU/l)**	58.6±11.59	61.6±15.03	62.54±14.61
	**GGT (IU/l)**	38.28±29.6	39.64±29.68	36.83±24.73

## References

[1] Meister A., Anderson M.E. (1983). Glutathione. Annu. Rev. Biochem..

[2] Jones D.P. (2006). Redefining oxidative stress. Antioxid. Redox Signal..

[3] Fang Y.-Z., Yang S., Wu G. (2002). Free radicals, antioxidants, and nutrition. Nutrition.

[4] Ketterer B., Coles B., Meyer D.J. (1983). The role of glutathione in detoxication. Environ. Health Perspect..

[5] Sen C.K. (1998). Redox signaling and the emerging therapeutic potential of thiol antioxidants. Biochem. Pharmacol..

[6] Meister A. (1994). Glutathione, ascorbate, and cellular protection. Cancer Res..

[7] Cruz R., Almaguer Melian W., Bergado Rosado J.A. (2003). Glutathione in cognitive function and neurodegeneration. Rev. Neurol..

[8] Smeyne M., Smeyne R.J. (2013). Glutathione metabolism and Parkinson’s disease. Free Radic. Biol. Med..

[9] Gul M., Kutay F.Z., Temocin S., Hanninen O. (2000). Cellular and clinical implications of glutathione. Indian J. Exp. Biol..

[10] Loguercio C., Taranto D., Vitale L.M., Beneduce F., Del Vecchio Blanco C. (1996). Effect of liver cirrhosis and age on the glutathione concentration in the plasma, erythrocytes, and gastric mucosa of man. Free Radic. Biol. Med..

[11] Herzenberg L.A., De Rosa S.C., Dubs J.G., Roederer M., Anderson M.T., Ela S.W., Deresinski S.C., Herzenberg L.A. (1997). Glutathione deficiency is associated with impaired survival in HIV disease. Proc. Natl. Acad. Sci. U.S.A..

[12] Giugliano D., Ceriello A., Paolisso G. (1995). Diabetes mellitus, hypertension, and cardiovascular disease: which role for oxidative stress?. Metabolism.

[13] Usal A., Acartürk E., Yüregir G.T., Unlükurt I., Demirci C., Kurt H.I., Birand A. (1996). Decreased glutathione levels in acute myocardial infarction. Jpn. Heart J..

[14] Viña J., Sastre J., Anton V., Bruseghini L., Esteras A., Asensi M. (1992). Effect of aging on glutathione metabolism. Protection by antioxidants. EXS.

[15] Jones D.P., Mody V.C., Carlson J.L., Lynn M.J., Sternberg P. (2002). Redox analysis of human plasma allows separation of pro-oxidant events of aging from decline in antioxidant defenses. Free Radic. Biol. Med..

[16] Zhang H., Forman H.J., Choi J. (2005). Gamma-glutamyl transpeptidase in glutathione biosynthesis. Methods Enzymol..

[17] Furukawa T., Meydani S.N., Blumberg J.B. (1987). Reversal of age-associated decline in immune responsiveness by dietary glutathione supplementation in mice. Mech. Ageing Dev..

[18] Schwartz J.L., Shklar G. (1996). Glutathione inhibits experimental oral carcinogenesis, p53 expression, and angiogenesis. Nutr. Cancer.

[19] Kim S.J., Han D., Ahn B.H., Rhee J.S. (1997). Effect of glutathione, catechin, and epicatechin on the survival of Drosophila melanogaster under paraquat treatment. Biosci. Biotechnol. Biochem..

[20] Allen J., Bradley R.D. (2011). Effects of oral glutathione supplementation on systemic oxidative stress biomarkers in human volunteers. J. Altern. Complement. Med..

[21] Witschi A., Reddy S., Stofer B., Lauterburg B.H. (1992). The systemic availability of oral glutathione. Eur. J. Clin. Pharmacol..

[22] Atkuri K.R., Mantovani J.J., Herzenberg L.A., Herzenberg L.A. (2007). N-Acetylcysteine—a safe antidote for cysteine/glutathione deficiency. Curr. Opin. Pharmacol..

[23] A.T.P. III At-A-Glance: Quick Desk Reference-NHLBI, NIH. 〈http://www.nhlbi.nih.gov/health-pro/guidelines/current/cholesterol-guidelines/quick-desk-reference-html〉 (accessed Feb 18, 2015).

[24] McPherson R.A., Hardy G. (2012). Cysteine: the Fun-Ke nutraceutical. Nutrition.

[25] Akerboom T.P., Sies H. (1981). Assay of glutathione, glutathione disulfide, and glutathione mixed disulfides in biological samples. Methods Enzymol..

[26] Ellman G.L. (1959). Tissue sulfhydryl groups. Arch. Biochem. Biophys..

[27] Riddles P.W., Blakeley R.L., Zerner B. (1983). Reassessment of Ellman’s reagent. Methods Enzymol..

[28] Handelman G.J., Machlin L.J., Fitch K., Weiter J.J., Dratz E.A. (1985). Oral alpha-tocopherol supplements decrease plasma gamma-tocopherol levels in humans. J. Nutr..

[29] Friedewald W.T., Levy R.I., Fredrickson D.S. (1972). Estimation of the concentration of low-density lipoprotein cholesterol in plasma, without use of the preparative ultracentrifuge. Clin. Chem..

[30] Moher D., Hopewell S., Schulz K.F., Montori V., Gøtzsche P.C., Devereaux P.J., Elbourne D., Egger M., Altman D.G. (2012). CONSORT CONSORT 2010 explanation and elaboration: updated guidelines for reporting parallel group randomised trials. Int. J. Surg..

[31] Bartoli G.M., Sies H. (1978). Reduced and oxidized glutathione efflux from liver. FEBS Lett..

[32] Li X., Qu Z.C., May J.M. (2001). GSH is required to recycle ascorbic acid in cultured liver cell lines. Antioxid. Redox Signal..

[33] Montecinos V., Guzmán P., Barra V., Villagrán M., Muñoz-Montesino C., Sotomayor K., Escobar E., Godoy A., Mardones L., Sotomayor P., Guzmán C., Vásquez O., Gallardo V., van Zundert B., Bono M.R., Oñate S.A., Bustamante M., Cárcamo J.G., Rivas C.I., Vera J.C. (2007). Vitamin C is an essential antioxidant that enhances survival of oxidatively stressed human vascular endothelial cells in the presence of a vast molar excess of glutathione. J. Biol. Chem..

[34] Halpner A.D., Handelman G.J., Harris J.M., Belmont C.A., Blumberg J.B. (1998). Protection by vitamin C of loss of vitamin E in cultured rat hepatocytes. Arch. Biochem. Biophys..

[35] Rushworth G.F., Megson I.L. (2014). Existing and potential therapeutic uses for N-acetylcysteine: the need for conversion to intracellular glutathione for antioxidant benefits. Pharmacol. Ther..

